# New Ground-Breaking Strategies in Bone Regeneration—In Memory of Nerio Ceroni

**DOI:** 10.3390/biomedicines10040855

**Published:** 2022-04-06

**Authors:** Alessandro De Vita, Davide Maria Donati, Laura Mercatali, Toni Ibrahim

**Affiliations:** 1 Osteoncology Unit, Bioscience Laboratory, IRCCS Istituto Romagnolo Per Lo Studio Dei Tumori (IRST) “Dino Amadori”, 47014 Meldola, Italy; laura.mercatali@irst.emr.it; 2 3rd Orthopaedic and Traumatologic Clinic Prevalently Oncologic, IRCCS Istituto Ortopedico Rizzoli, 40136 Bologna, Italy; davidemaria.donati@ior.it; 3 Osteoncology, Bone and Soft Tissue Sarcomas and Innovative Therapies Unit, IRCCS Istituto Ortopedico Rizzoli, 40136 Bologna, Italy; toni.ibrahim@irst.emr.it

This editorial article is dedicated to the memory of the Nerio Ceroni, the grandfather of the first author. Nerio Ceroni suffered from bone metastases and passed away in January 2022.

The complex biological mechanisms which orchestrate bone homeostasis are represented by a balance between bone formation and bone resorption, which persists for all of life. In this regard, dysregulation of these physiological processes is present in a variety of pathological conditions, including bone defects created by trauma, infections, tumor resections, skeletal-related events and abnormalities, vascular necrosis, and osteoporosis. Great efforts have been made to dissect the biological interplay that subsists between the different bone-related cells and to improve the therapeutic strategies to restore skeletal functions. Articles published as part of the Special Issue “New Ground-Breaking Strategies in Bone Regeneration—in Memory of Nerio Ceroni” highlight the recent advances in this field, as well as emerging strategies to enlarge the therapeutic scenario for patients suffering from bone-related diseases.

Bone defect models represent promising tools for improving the current understanding of bone disease pathophysiology and may provide useful information about the functional and mechanical properties of implants. In this regard, the animal models in current use often fail to reproduce the full spectrum of mechanical functions of bone impaired by a defect and restored during skeletal reconstitution. Moreover, the study of plates implantation or loadbearing bone substitutes is not always permitted. For the above reasons, there is a pressing need to develop new models to overcome these drawbacks. Baskin and colleagues developed an animal model for in vivo mechanical strength testing using a specific surgical procedure to create defects enabling the preservation of nerves, vascular and dental tissue in the anterior ramus of the rat and rabbit mandible [1]. This pilot study allowed the production of in vivo biomechanical information through the use of animals with elodont dentition and unfused mandible symphyses, which are hypothesized to have symmetric incisor morphology. Furthermore, the explored defects allowed the authors to observe a relationship between hemi-mandible stability and ipsilateral tooth growth. This provides a starting point for further research aimed at validating these innovative models in bone regeneration panorama.

As described above, bone mechanobiology holds a pivotal role in fracture consolidation. Barcik et al. investigated the impact of mechanical stimulation on the short-term response of healing tissue. In particular, they assessed the progression of healing during stimulation and resting phases in four sheep with tibial osteotomy. The real-time measurement of tissue stiffness allowed the authors to observe the average daily stiffness increase during the resting period compared to periods of stimulation for all the included sheep. These preliminary data suggested that resting periods represent a crucial factor in bone healing, improving tissue stiffness and active fixation [2]. Since mechanical stimulation is known to promote callus formation and secondary bone healing, the data presented on sheep models partially contradicted this assumption. Therefore, this poses the question of the real clinical benefit impact of mechanical stimulation in bone repair and musculoskeletal rehabilitation. This is what Barcik et al. reported in their review [3], which investigated whether optimization of the mechanical environment over the course of bone regeneration could impact the shortening of bone healing time. In an attempt to answer this question, the authors found that translational studies on the course of fracture healing reported that both resting period and application of mechanical stimulation had an impact on the different phases of bone growth from injury. In this regard, they suggested that mechanical stimulation is not required in the inflammation phase, while it could promote the growth of callus tissue adjacent to the fracture during the subsequent proliferation phase compared to unstimulated tissues. The following phases are represented by consolidation, in which constant stimulation seems to fail tissue consolidation, and remodeling in which most studies provide anecdotal insight about optimum mechanical conditions. On the basis of the obtained observation, the authors formulated a hypothesis for the stimulation protocol which could shorten the healing time using stimulation predominantly in the proliferative phases and resting periods between the applications of mechanical stimulation.

In the landscape of research on bone-related diseases, bone-tissue engineering represents an important field of application. Treatment of bone defects is still a major clinical problem and intensive research is being dedicated to improve the current daily clinical management of such lesions. In this regard, 3D biomimetic scaffolds are widely studied, together with autologous or heterologous bone grafts, for the treatment of bone defects. Ruffini and colleagues provide a review [4] on the use of ion-doped apatites as biomimetic materials obtained through novel nature-inspired protocols which preserve their chemical properties, allowing the generation of advanced biofunctional properties including biomorphic transformations. This emerging approach based on the complete chemical conversion of woods into bone scaffolds maintaining biomorphic and hierarchical architecture represents a key enabling technology, especially for critical-size and nonunion defects in long bone and potential application in different other pathological conditions.

In the same field of tissue engineering and materials science, Dubnika et al. reported the development of 3D autologous platelet-rich fibrin (PRF) matrices as innovative drug-delivery systems of vancomycin hydrochloride using liposomes and microparticles [5]. They reported the physicochemical characterization of these nano- and micro-carriers, furthermore, they provided insights into the scaffold structure through the use of microtomography. Finally they assessed the antibacterial activity of the devices on a bacterial suspension of staphylococcus aureus. The results showed the ability of these smart materials to achieve a complete antibacterial effect for 48 h and provide proof of concept of their promising role in tissue engineering.

Primary and metastatic bone tumors represent a variety of solid lesions affecting the patient’s quality of life with important comorbidities. In this regard, bone sarcomas constitute a highly heterogeneous group of rare mesenchymal primary neoplasms of bone. They are characterized by a relatively high morbidity and mortality, especially in children and adolescent patients. Among them, giant cell tumor of bone (GCTB) and desmoplastic fibroma (DF) are bone sarcomas with locally aggressive behavior and unpredictable prognosis. They exhibit a predilection for the long bone or mandible of young adults and are responsible for severe bone disruption. The current pathological mechanism of osteoclastogenesis processes in these two bone primary tumors is still not well understood. The current treatment strategies include surgery, radiotherapy and chemotherapy, but the benefit of the latter is yet to be completely elucidated. In order to improve the current understanding of these poorly investigated diseases, De Vita and colleagues shed light on the molecular biology of these lesions. Moreover, the authors proposed the combination of bone target therapy drug denosumab and an innovative multitarget tyrosine kinase inhibitor, Lenvatinib, for the treatment of bone sarcomas including giant cell tumor of bone and desmoplastic fibroma [6]. By taking advantage of the combination of a win–win approach using patient-derived primary cultures, 3D culture systems and in vivo zebrafish analysis in zebrafish xenotransplanted bone sarcoma models they provide new insight on the involvement of some bone- and vasculature-related biomarkers and the rationale for deepening this drug combination in the clinical setting.

Future directions in bone regeneration will include an integrated approach exploiting tissue engineering technologies with the use of autologous or allogeneic bone grafts and bone-graft substitutes, combined with mesenchymal stem cells, osteoprogenitors, gene therapy and mechanical stability devices.

In conclusion, bone-related diseases are a complex spectrum of malignancies with challenging clinical management, important patient outcomes and socioeconomic impact. Great efforts are being devoted to improving the patient quality of life and to solve some of the clinical needs. A multidisciplinary approach in this research field could pave the way for establishing new therapeutic protocols and treatment strategies for patients affected by bone-related diseases.
